# Brain functional connectivity in children with developmental coordination disorder following rehabilitation intervention

**DOI:** 10.1038/s41390-021-01517-3

**Published:** 2021-05-01

**Authors:** Sara Izadi-Najafabadi, Shie Rinat, Jill G. Zwicker

**Affiliations:** 1 Graduate Programs in Rehabilitation Sciences, University of British Columbia, Vancouver, Canada; 2 BC Children’s Hospital Research Institute, Vancouver, Canada; 3 Department of Occupational Science & Occupational Therapy, University of British Columbia, Vancouver, Canada; 4 Department of Pediatrics, University of British Columbia, Vancouver, Canada; 5 Sunny Hill Health Centre at BC Children’s Hospital, Vancouver, Canada; 6 CanChild Centre for Childhood Disability Research, Hamilton, Canada

## Abstract

**Background:**

Children with developmental coordination disorder (DCD) show improved motor function after Cognitive Orientation to Occupational Performance (CO-OP) intervention; however, the neural basis for these improvements is unknown.

**Methods:**

In this randomized waitlist-controlled trial, 78 children with DCD (with/without ADHD) were randomly assigned to either a treatment or waitlist group and underwent three resting-state MRI scans over six months. The treatment group received intervention between the first and second scan; the waitlist group received intervention between the second and third scan.

**Results:**

After CO-OP intervention, children with DCD [13 male, 8 female; mean (SD) age: 10.0 (1.7) years] showed increased functional connectivity between the default mode network and right anterior cingulate gyrus (*p* < 0.01). Additional gains were noted at follow-up three months after the intervention, with greater functional connectivity between the dorsal attention network and precentral gyrus (*p* < 0.02). However, children with DCD + ADHD [18 male, 1 female; mean (SD) age: 10.0 (1.14) years] did not show brain changes following CO-OP.

**Conclusion:**

For children with DCD, increased functional connectivity in networks associated with self-, emotion-, and attention-regulation may underlie motor skill improvements observed after CO-OP intervention. Modifications to the CO-OP protocol may be required to induce similar brain changes in children with DCD + ADHD.

**Impact:**

- This study provides neuroscientific evidence for the Cognitive Orientation to Occupational Performance (CO-OP) approach as an effective rehabilitation intervention to induce brain and behavioral changes in children with DCD.
- While children with DCD ± ADHD showed improved motor function after CO-OP, only children with DCD showed brain changes after intervention.
- Children with DCD showed increased functional connectivity in networks associated with self-, emotion-, and attention-regulation after the intervention.
- Treatment modifications may be required to induce similar brain changes in children with DCD + ADHD.
- Pediatricians are encouraged to refer children with DCD  with and without ADHD for CO-OP intervention to improve their motor skills.

## Introduction

Affecting one in 20 children, developmental coordination disorder (DCD) is a chronic disorder of unknown etiology that affects a child’s ability to learn motor skills and participate in daily tasks, leisure activities, and play^1^. Up to 50% of children with DCD also have co-occurring attention deficit hyperactivity disorder (ADHD)^2,3^, which further exacerbates motor and functional issues^2,4^.

Several neuroimaging studies have shown that children with DCD exhibit brain differences compared to typically-developing (TD) children in the cerebellum, basal ganglia, corpus callosum, parietal lobe, and part of the frontal lobe^5,6^. While knowledge of these brain sources of DCD has been beneficial to better understand the nature of this disorder, it is important to understand if rehabilitation intervention can induce neuroplastic change and improve outcomes. In a recent systematic review of rehabilitation-induced changes on MRI in children with neurodevelopmental disorders (i.e., ADHD, autism spectrum disorder, cerebral palsy, fetal alcohol spectrum disorder, learning disorders), we did not find any studies that included children with DCD ± ADHD^7^. A few studies have investigated training-induced brain plasticity in children with DCD, but these studies did not include response to rehabilitation intervention^8^. Findings consistently reported brain changes in the frontal and parietal lobes associated with an overload of attentional resources and cognitive fatigue during motor learning and automatization^8^.

According to international clinical practice guidelines, one of the recommended treatments for children with DCD is a rehabilitation approach known as Cognitive Orientation to Occupational Performance (CO-OP)^9^. It is an individualized, client-centered intervention primarily designed for children with DCD to improve what they want or need to do in everyday life^10,11^. It is a cognitive-based, problem-solving approach that uses verbal mediation and identifies cognitive strategies to influence functional motor skill acquisition. While the CO-OP approach has been effective in meeting motor goals of children with DCD^12,13^ and ADHD^13,14^, the underlying mechanism and the neural basis for these improvements are unknown. A better understanding of the underlying mechanisms of CO-OP could be used to optimize CO-OP’s effectiveness or modify it to meet the needs of the target population. Therefore, the goal of this study was to examine neuroplastic changes in whole-brain functional connectivity associated with rehabilitation intervention in children with DCD ± ADHD.

## Method

### Study design

This study is part of a randomized waitlist-controlled trial (ClinicalTrials.gov ID: NCT02597751) comparing brain structure and function of children with DCD, DCD + ADHD, and TD children longitudinally. This paper focuses on the intervention portion of the trial where children with DCD and DCD + ADHD were randomly assigned to either a treatment or waitlist group and changes in resting-state MRI were examined. A statistician prepared the randomization sequence of participants using computer-generated sequential blocks of 4–6; the study team was blinded to group allocation until after enrollment and the first MRI scan (opaque-sealed envelope concealment). To provide 80% power to detect a clinically significant improvement of 2 points on the primary outcome measure [Canadian Occupational Performance Measure (COPM)]^15^ with a standard deviation of 2.5 and a type-1 error of 0.05, 25 participants per group were required. To ensure sufficient power for our neuroimaging analyses, we used our pilot study on diffusion tensor imaging in this population^16^ to estimate that a sample size of 30 per group would detect a 3% difference in axial diffusivity. Secondary outcomes measures included the Performance Quality Rating Scale (PQRS)^17^ to evaluate movement quality and the Bruininks–Oseretsky Test of Motor Proficiency—second ed. (BOT-2)^18^ to measure overall motor ability. A schematic of the study design^8^, inclusion criteria, detailed description of assessment tools, and behavioral outcomes^13^ have been reported elsewhere. This study was approved by the University of British Columbia/Children’s and Women’s Health Center of British Columbia Research Ethics Board. Parents consented and children assented to participate in the study.

### Participants

**Fig. 1:**
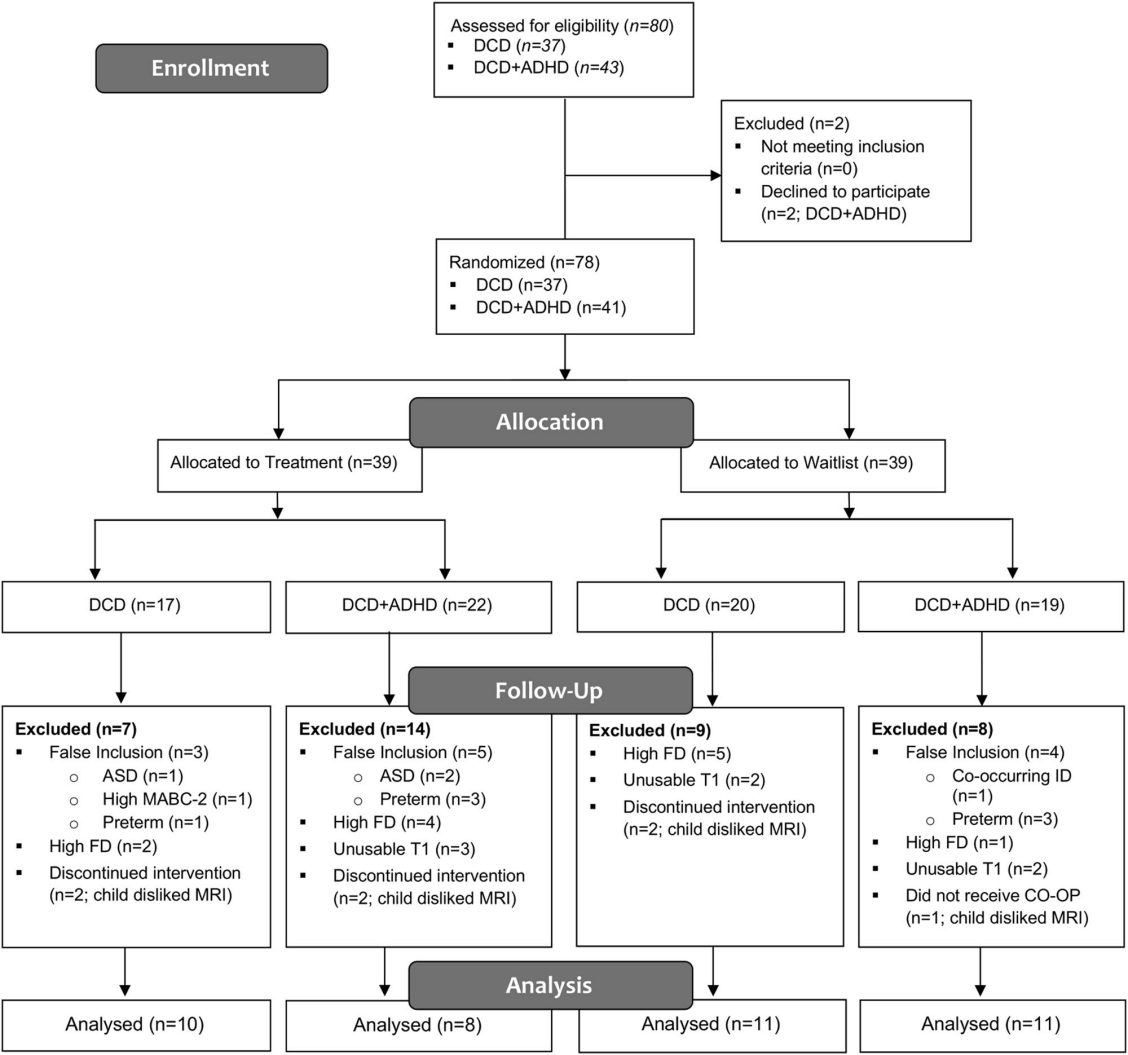
CONSORT flow diagram. ADHD attention deficit hyperactivity disorder, ASD autism spectrum disorder, CO-OP Cognitive Orientation to Occupational Performance, DCD developmental coordination disorder, FD framewise displacement, ID intellectual disability, MABC-2 Movement Assessment Battery for Children— second edition.

Using a sample of convenience, a total of 80 children (37 children with DCD and 43 children with DCD + ADHD) were recruited from Dr. Zwicker’s research-integrated DCD Clinic at Sunny Hill Health Center for Children, BC Children’s Hospital ADHD Clinic, or from the community (Vancouver, BC) from September 2014 to July 2019. Seventy-eight children—37 with DCD (25 male, 12 female) and 41 with DCD + ADHD (38 male, 3 female)—met the inclusion criteria and were randomized to treatment and waitlist groups. Enrolled children first went through an MRI safety screening and MRI simulation session to get familiarized with the task and alleviate their anxiety. A research nurse and graduate students scanned children using MRI at baseline, after three months, and after 6 months. Children in the treatment group received intervention between the first and second MRI scans, while children in the waitlist group received intervention between the second and third MRI scans. Thirty-eight participants (20 DCD and 18 DCD + ADHD) were excluded from the analysis (see Fig. 1 for details). Overall, we analyzed pre-post data from 21 children with DCD and 19 children with DCD + ADHD (Table 1). For analyses related to the maturation effect and follow-up, we were limited to data from eight participants due to the quality of baseline or follow-up scans. One child with DCD and 11 children with DCD + ADHD took ADHD-related stimulant medications (e.g., methylphenidate, lisdexamfetamine, dextroamphetamine/amphetamine) at the time of intervention; medication was used as a covariate in the analysis.

**Table 1:** Participant characteristics and head motion parameters.

Variable		DCD (*n* = 21)		DCD + ADHD (*n* = 19)	
		Treatment (*n* = 10)	Waitlist (*n* = 11)	Treatment (*n* = 8)	Waitlist (*n* = 11)
*Participant characteristics*					
Male Sex assigned at birth; *N* (%)		7 (70)	6 (54)	8 (100)	10 (91)
Age (years); Mean (SD)		10.8 (1.7)	9.3 (1.5)	10.1 (1.1)	9.9 (1.2)
DCDQ (total); Mean (SD)		26.0 (4.9)	36.3 (9.6)	28.1 (12.5)	33.6 (6.9)
MABC-2 (percentile); Median (IQR)		2 (4)	2 (5.8)	0.75 (2.4)	9 (11.8)
Conner’s ADHD Index (T-score); Median (IQR)		90 (13.8)	81 (26.5)	90 (4.3)	90 (0)
*Head motion parameters*					
Framewise Displacement (mm); Mean (SD)	Scan 1	0.18 (0.10)	0.33 (0.28)	0.24 (0.13)	0.23 (0.15)
	Scan 2	0.14 (0.11)	0.20 (0.13)	0.24 (0.17)	0.20 (0.12)
	Scan 3	0.19 (0.16)	0.20 (0.15)	0.23 (0.14)	0.22 (0.13)
Relative displacement (mm); Mean (SD)	Scan 1	0.10 (0.06)	0.17 (0.17)	0.13 (0.07)	0.13 (0.09)
	Scan 2	0.10 (0.09)	0.10 (0.08)	0.13 (0.10)	0.11 (0.07)
	Scan 3	0.11 (0.09)	0.11 (0.09)	0.12 (0.08)	0.12 (0.08)
Absolute displacement (mm); Mean (SD)	Scan 1	0.40 (0.27)	0.76 (0.74)	0.52 (0.46)	0.50 (0.36)
	Scan 2	0.32 (0.20)	0.37 (0.30)	0.53 (0.39)	0.50 (0.30)
	Scan 3	0.80 (1.10)	0.50 (0.36)	0.56 (0.49)	0.67 (0.64)

*ADHD* attention deficit hyperactivity disorder, *DCD* developmental coordination disorder, *DCDQ* Developmental Coordination Disorder Questionnaire, *IQR* inter-quartile range, *MABC-2* Movement Assessment Battery for Children—second ed., *SD* standard deviation.

### Intervention

Each child identified three functional goals (e.g., handwriting, playing basketball, tying shoelaces) on which to work during the intervention. Registered occupational therapists administered one-hour of CO-OP intervention once weekly for 10 weeks for each child as per published protocol^10^. As part of the intervention [described in detail elsewhere^13^], therapists guide children to discover cognitive strategies to solve their motor problems and learn motor skills^10^. Parents also received training to apply CO-OP strategies at home to facilitate additional practice and generalization and transfer to other motor skills.

### Data analysis

Wilcoxon Signed-Rank Tests were used to compare self-perceived motor performance and satisfaction as well as movement quality and overall motor ability before and after CO-OP intervention. *R*-value effect sizes were also calculated. Alpha was set at 0.05 and corrected for multiple testing using Bonferroni; statistical significance was considered *p* < 0.004.

### MRI protocol

In this study, all MRI data were acquired on a 3-Tesla General Electric Discovery MR750 MRI scanner using a 32-channel head coil. During resting-state MRI acquisition, participants were asked to lie very still and to not think of anything while being scanned. At least one 5-min resting-state functional MRI gradient-recalled echo planar imaging sequence (TR = 3000 ms, TE = 30 ms, FOV = 288, acquisition matrix = 96 × 96, flip angle = 90°, number of slices = 52, slice thickness = 3 mm) was acquired and repeated in case of participant movement. One high-resolution 3D T1 anatomical image was taken for registration purposes (3D FSPGR, TR = 8180 ms,  TE = 3192 ms, FOV = 256, acquisition matrix = 256 × 256, flip angle = 12°, number of slices = 188, slice thickness = 1 mm).

### Brain imaging analyses

#### T1-weighted Images

T1-weighted images were visually inspected for motion artifacts. Seven participants were excluded due to low-quality scans from excessive head motion. Brain extraction was performed using FreeSurfer (v5.3.0)^19^.

#### Resting-state functional MRI

Resting-state functional MRI data were only included if the framewise displacement (FD) was less than 0.5 mm (Table 1); FD indexes head movement and changes in head position from one frame to the next^20^. Accordingly, data obtained from 12 participants were excluded from analysis due to high FD. We used the FMRIB Software Library (FSL) for all steps of the analysis^21^. Pre-processing steps included MCFLIRT motion correction, slice timing correction, and high-pass filtering at the cut-off of 0.01 Hz. Further, denoising was performed using MELODIC independent component analysis (ICA) and FMRIB’s ICA-based Xnoiseifier (FIX; e.g., location, size, power spectra, and time-series)^22^. Hand-classifications of components by the first and second authors for 20 participants were used to train FIX and allowed the automated classification process and soft clean-up with 24 motion confound regression. Following FIX denoising, we further cleaned our data using white matter and cerebrospinal fluid signal regression through CONN functional connectivity toolbox^23^. Pre-processing was completed by spatial smoothing (6 mm full width and half maximum) and registering resting-state functional images to the standard template (MNI 152 2 mm).

**Fig. 2:**
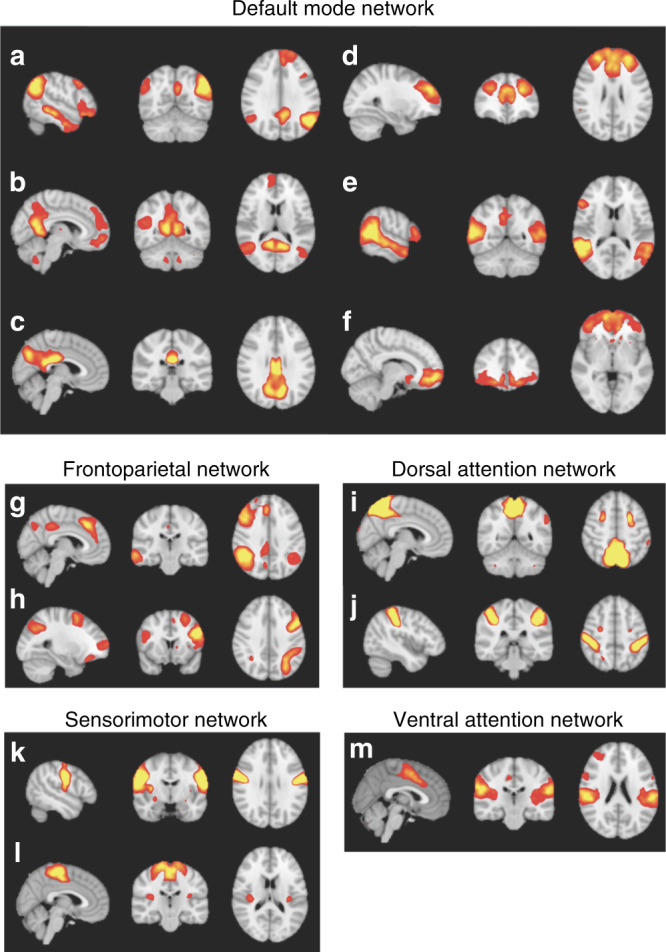
Thirteen independent components and five resting-state networks from the current study thresholded at *z* > 5. Default mode network [six independent components: (**a**), (**b**), (**c**), (**d**), (**e**), (**f**)); frontoparietal network (two independent components: (**g**), (**h**)); dorsal attention network (two independent components: (**i**), (**j**)); sensorimotor network (two independent components: (**k**), (**l**)); ventral attention network (one independent component: (**m**)].

Group-level ICA with 25 components was conducted to temporally concatenate data (*n* = 151) across all participants (children with DCD, children with DCD + ADHD, and TD children) and all sessions (Scan 1–3), to identify resting-state networks in our data. We calculated Pearson’s *r* between the spatial maps of our group-level ICA components and Yeo networks^24^, a set of seven popular resting-state networks. We excluded components with low spatial correlation (*r* < 0.204) with Yeo networks as well as all components correlating with the visual network, as we were unable to control whether children kept their eyes open or closed. Thirteen components forming sensorimotor, dorsal attention networks (DAN), ventral attention, frontoparietal, and default mode networks (DMN)^24^ were put forward for dual-regression and statistical analysis (Fig. 2).

Dual regression results were fed into permutation analysis of linear models (PALM)^25^ with 5000 permutations to compare functional connectivity over three months of maturation (scan 1 and scan 2 of waitlist groups), before and after CO-OP intervention (pre- and post-intervention scans of both treatment and waitlist groups), and after three months of follow-up (scan 2 and scan 3 of treatment group) using paired *t*-tests, controlling for the effect of ADHD-related medications^26–29^ on the brain. We also used PALM to investigate the relationship of motor outcomes (PQRS)^17^ and Bruininks–Oseretsky Test of Motor Proficiency—second ed. (BOT-2)^18^ with functional connectivity in the two groups. The results were thresholded using threshold-free cluster enhancement (TFCE) and were corrected for contrasts and for multiple testing using family-wise error correction (FWE) with an alpha level of 0.05 and a minimum cluster size of five voxels. TFCE retains spatial details of extended signals in a cluster-like area, which makes it a more sensitive thresholding approach compared to voxel or cluster-based thresholding^30^. The Harvard-Oxford cortical atlas was used to identify brain regions^31^.

## Results

### Behavioral results

After CO-OP intervention, children with DCD and children with DCD + ADHD showed statistically significant (*p* < 0.001) improvement in their perceived motor performance and satisfaction on their motor goals [as measured by the COPM^15^] and in observed movement quality [as measured by the PQRS^17^]. While both groups showed improved scores on the BOT-2^18^, these findings were not significant after Bonferroni correction (Table 2).

**Table 2:** Motor outcomes before and after CO-OP intervention.

	DCD (*n* = 21)		*p*	*r*	DCD + ADHD (*n* = 19)		*p*	*r*
	Pre-test Median (IQR)	Post-test Median (IQR)			Pre-test Median (IQR)	Post-test Median (IQR)		
COPM Performance	2.7 (1.8)	6.7 (1.6)	**<0.001**	0.62	2.3 (1.6)	7.0 (1.2)	**<0.001**	0.60
COPM Satisfaction	3.0 (2.3)	8.0 (1.5)	**<0.001**	0.62	2.3 (3.3)	8.0 (2.0)	**<0.001**	0.60
PQRS	3.0 (1.9)	6.3 (1.8)	**<0.001**	0.61	3.0 (1.7)	5.7 (2.3)	**<0.001**	0.60
BOT-2 (percentile)	12 (15.5)	16 (21.5)	0.02	0.35	12 (25)	21 (39)	0.005	0.45

Bonferroni-corrected, significant *p*-values (*p* < 0.004) are bolded.

*ADHD* attention deficit hyperactivity disorder, *BOT-2* Bruininks–Oseretsky Test of Motor Proficiency—second edition, *COPM* Canadian Occupational Performance Measure, *DCD* developmental coordination disorder, *IQR* inter-quartile range, *PQRS* Performance Quality Rating Scale.

### Brain imaging results: DCD-only group

#### Maturation

**Fig. 3:**
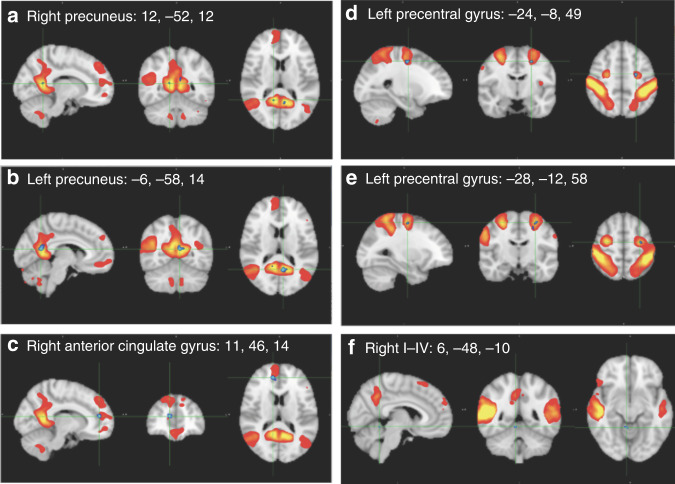
Functional connectivity in children with DCD. Effect of maturation (**a**), (**b**), intervention (**c**), follow-up (**d**), (**e**) on functional connectivity, Relationship of functional connectivity with movement quality (**f**). Group-level ICA spatial maps are shown in red-yellow and clusters showing a significant change in functional connectivity are in blue.

Comparing functional connectivity between the first and second scans of eight children with DCD in the waitlist group showed a significant increase (FWE-*p* < 0.03) in functional connectivity between right/left precuneus (Table 3 and Fig. 3a, b) and the DMN. Regions of this component of the DMN include the right/left precuneus, middle and superior frontal gyrus, frontal pole, right/left lateral occipital cortex, left parahippocampal gyrus, temporal fusiform, middle temporal gyrus, and cerebellar lobules of left VIIb, IX, and crus II.

**Table 3:** Functional connectivity in children with DCD: effect of maturation, intervention, and follow-up and relationship with motor outcomes^a^.

Network	Region	MNI-space			*t*	FWE-*p*	Cluster size^b^	Cohen’s d
		*x*	*y*	*z*				
*Maturation*								
DMN	L Precuneus	−6	−58	14	10.01	0.01	96	2.7
DMN	R Precuneus	12	−52	12	14.16	0.03	6	3.8
*Intervention effect*								
DMN	R Anterior Cingulate Gyrus	11	46	14	6.40	0.01	44	1.02
*Follow-up*								
DAN	L Precentral Gyrus	−24	−8	49	7.31	0.02	20	1.83
DAN	L Precentral Gyrus	−28	−12	58	6.13	0.03	15	1.53
*Motor outcomes (PQRS)*								
DMN	R Cerebellar Lobules I–IV	6	−48	−10	5.90	0.03	10	0.53

*DAN* dorsal attention network, *DCD* developmental coordination disorder, *DMN* default mode network, *FWE* family-wise error corrected, *L* left, *PQRS* Performance Quality Rating Scale, *R* right.

^a^Effects are shown with threshold-free cluster enhancement (TFCE) and a minimum cluster size of five voxels.

^b^Number of voxels (voxel size = 2 mm).

#### Intervention effect

Comparing pre- and post-intervention functional connectivity of 21 children with DCD showed a significant increase (FWE-*p* < 0.01) in functional connectivity of the DMN with the right pregenual anterior cingulate gyrus (Brodmann areas 32 and 24; Table 3 and Fig. 3c) after CO-OP intervention.

#### Follow-up

Comparing the second and third scans of eight children with DCD in the treatment group (measuring functional connectivity three months after completing CO-OP intervention) indicated a significant increase in the functional connectivity of the DAN and left precentral gyrus (Table 3 and Fig. 3d, e). This component of the DAN is comprised of the precuneus, middle and superior frontal gyrus, frontal operculum, precentral gyrus, cingulate cortex, lingual gyrus, right caudate, and temporal fusiform cortex.

#### Relationship of motor outcomes and functional connectivity

Regression analysis showed that higher PQRS scores significantly (FWE-*p* < 0.05) predicted greater functional connectivity between the DMN (Fig. 2e) and right cerebellar lobules I–IV (Table 3 and Fig. 3f) in children with DCD. Brain regions in this component of the DMN are comprised of the right middle temporal gyrus, angular gyrus, precuneus, superior frontal gyrus, frontal orbital cortex, temporal fusiform cortex, and left crus II.

### Brain imaging results: DCD + ADHD group

Children with DCD + ADHD did not show any significant change (FWE-*p* > 0.05) in functional connectivity in the three-month period before the CO-OP intervention (*n* = 10), immediately after CO-OP intervention (*n* = 19), or in the three-month follow-up analysis (*n* = 7).

#### Relationship of motor outcomes and functional connectivity

**Fig. 4:**
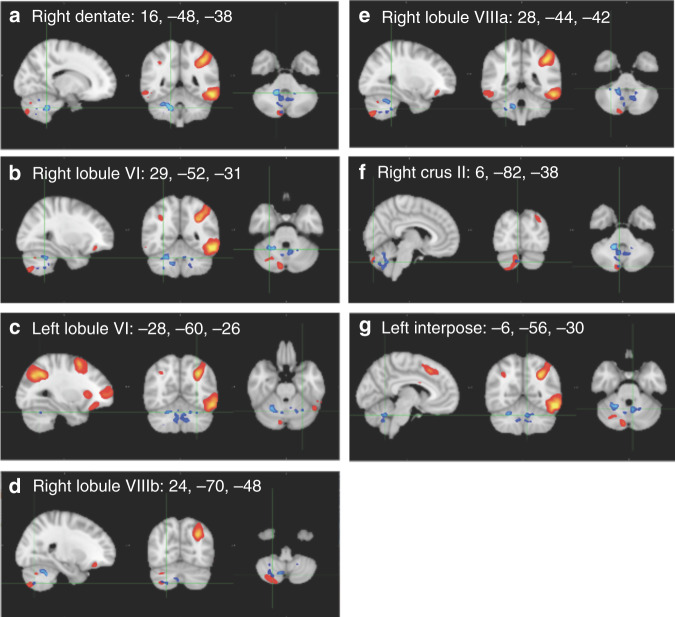
Functional connectivity in children with DCD + ADHD. Relationship of functional connectivity and movement quality (**a**)–(**g**). Group-level ICA spatial maps are shown in red-yellow and clusters showing a significant change in functional connectivity are in blue.

Pre- and post-intervention PQRS scores significantly predicted functional connectivity between the frontoparietal network (Fig. 2h) and seven cerebellar regions (Table 4 and Fig. 4a–h): right dentate, right and left lobule VI, right lobule VIIIa and VIIIb, right crus II, and left interpose. Brain regions of this frontoparietal network component include the left frontal pole, left supramarginal gyrus, left and right inferior temporal gyrus, right inferior frontal gyrus, left paracingulate gyrus, left caudate, left cingulate gyrus, right orbitofrontal cortex, angular gyrus, and right cerebellar Crus II.

**Table 4:** Functional connectivity in children with DCD + ADHD: relationship with motor outcomes^a^.

Network	Region	MNI-space			*t*	FWE-*p*	Cluster size^b^	Cohen’s d
		*x*	*y*	*z*				
*Motor outcomes (PQRS)*								
Frontoparietal	R Dentate	16	−48	−38	7.13	0.01	546	1.12
	R Lobule VI	29	−52	−31	3.52	0.03		0.56
Frontoparietal	L Lobule VI	−28	−60	−26	5.96	0.05	11	0.94
Frontoparietal	R Lobule VIIIb	24	−70	−48	4.66	0.04	46	0.73
Frontoparietal	R Lobule VIIIa	28	−44	−42	5.12	0.04	29	0.81
Frontoparietal	R Crus II	6	−82	−38	5.26	0.04	22	0.83
Frontoparietal	L interpose	−6	−56	−30	6.01	0.02	218	0.95

*ADHD* attention deficit hyperactivity disorder, *DCD* developmental coordination disorder, *FWE* family-wise error corrected, *L* left, *PQRS* Performance Quality Rating Scale, *R* right.

^a^Effects are shown with threshold-free cluster enhancement (TFCE) and a minimum cluster size of five voxels.

^b^Number of voxels (voxel size = 2 mm).

## Discussion

In this RCT, we used resting-state MRI to longitudinally assess brain changes associated with CO-OP intervention in children with DCD ± ADHD. Results showed that CO-OP intervention improved motor performance and movement quality in both groups of children. After the intervention, changes in functional connectivity between the DMN and the right anterior cingulate cortex (ACC) were noted in children with DCD. These changes are different from brain maturation over the same time period of three months in the DMN and the bilateral precuneus. Our results also suggest that CO-OP-induced changes in functional connectivity were retained three months after the intervention. Moreover, we captured a significant increase in the functional connectivity of the DAN and left precentral gyrus in the follow-up scans in children with DCD. However, children with a dual diagnosis of DCD and ADHD did not show any changes in brain functional connectivity following CO-OP intervention. In what follows, we discuss the effects of maturation, CO-OP intervention, and follow-up effects in more detail.

### Maturation

Children with DCD in the waitlist group showed increased functional connectivity between the DMN and the precuneus during the first three months of study, consistent with reports of DMN maturation^32,33^. We conducted this analysis to ensure that the changes in functional connectivity with the intervention were not due to brain maturation over the same time period.

### Intervention effect

We found that the DMN and the right pregenual anterior cingulate cortex (pACC) become more functionally connected after CO-OP intervention in children with DCD, which was unrelated to maturation. The pACC plays a crucial role in the cognitive regulation of emotion^34^, self-reflection^35,36^, social processing^37^, conflict-monitoring^38^, and inhibition of action^34,39^. Improved pACC functional connectivity and its role in emotion regulation are in line with our previous findings of improved white matter structure of the anterior thalamic radiation after CO-OP intervention^8^. Both the ACC and the anterior thalamic radiation are components of the Papez circuit facilitating emotion regulation^40^; the Papez circuit connects the anterior thalamic nuclei to the ACC through thalamic radiations and then travels back to the ACC through the parahippocampal gyrus and hippocampus^40^.

The pACC is located at the rostrum of the DMN where it works with the ventromedial prefrontal cortex to guide self-regulation^37,41–43^, problem-solving^38^, and internally-directed cognition^44^. Children with DCD experience difficulty with self-regulation^45–47^ and have shown atypical function and structure of the ACC^48–50^ and other regions of the DMN^49,51–57^. During CO-OP intervention, children with DCD acquire self-regulatory skills (e.g., goal-setting, planning, self-monitoring, evaluating) to address their motor performance difficulties^58,59^. Given the role of the ACC and DMN in self-regulation^37,41^, we infer that they may act as a self-regulatory system for children with DCD^58,59^. This is in line with studies on self-regulation in individuals with other neurodevelopmental^60,61^ or psychiatric diagnoses^62^ showing engagement of the ACC and the DMN.

The DMN is activated during rest as well as during an internally-directed task (e.g., thought, memories, mental imagery, envisioning immediate future); however, the DMN is deactivated when attending to external environment stimuli^44,63^. Increased functional connectivity of the DMN may enable children with DCD to regulate their attentional and cognitive resources^64,65^ to guide processes (e.g., internal, self-referential thoughts) other than motor tasks, which, in turn, can guide self-regulatory processes required for motor performance. Therefore, increased functional connectivity of the DMN observed during motor tasks^51^ or after CO-OP intervention in children with DCD might be a compensatory mechanism to engage internally-directed thoughts and guide self-regulation.

Consistent with our previous results^8^, all the observed changes in this study were located on the right hemisphere, reflecting its lateralization in the early stages of learning^66^, problem-solving, and emotion regulation^67^. Blais and colleagues also reported that early stages of bimanual motor learning and its attentional requirement could result in higher intra-hemisphere coherence in the right hemisphere in children with DCD^66^.

Unlike children with DCD and despite improved motor function, children with co-occurring DCD and ADHD did not show any brain changes associated with CO-OP intervention. Children with a dual diagnosis of DCD and ADHD experience more severe motor problems than children with DCD alone^2,4^, causing greater functional limitations and reduced social participation^68,69^. Moreover, they show different brain function^50,70,71^ and structure^48,72^ when compared to children with a single diagnosis of DCD or ADHD. Importantly, self-regulation, which seems to guide CO-OP’s mechanism of change^58,59^, is impaired in children with ADHD^73^. Taken together, we believe that the uniqueness of the brains of children with DCD and ADHD^48,50,70–72^ and their more severe functional^2,4^ and self-regulation difficulties^73^ may explain why CO-OP intervention did not induce similar effects to that of children with DCD only.

Exacerbated motor and functional difficulties in children with a dual diagnosis of DCD and ADHD^2,4^ are related to significant problems with attention in comparison to DCD only^74^. Accordingly, we can infer that children with DCD and ADHD may require higher self- and attention-regulation in order to induce long-lasting effects and brain changes. Evidence suggests that this may be feasible through modifications to the CO-OP protocol^14^ or combining CO-OP with medication or other self-regulatory interventions^75^. For example, in their pilot study of children with ADHD, Gharebaghy et al.^1^ suggest that the provision of more rest time and free play during CO-OP may be required for children with ADHD. Additional modifications, such as a longer intervention period, higher intensity, and more structured in-home practice may also help to improve the effectiveness of CO-OP intervention for children with DCD and co-occurring ADHD. Self-regulatory interventions for children with ADHD have an additional component of providing external feedback on the accuracy of self-monitoring and self-reinforcement, which can be added to CO-OP for children with DCD + ADHD to increase its effectiveness^75^.

### Three-month follow-up effect

Similar to motor performance^13^, children with DCD maintained their CO-OP-induced brain changes (i.e., increased functional connectivity of the DMN and the ACC) and developed higher within-network functional connectivity of the DAN and left precentral gyrus three months after the completion of CO-OP intervention. The DAN is known to mediate voluntary goal-driven attention and orient attention to cues^76,77^. In other words, the DAN plays a role in determining where, when, or to what participants direct their attention^76,78^. Considering that children with DCD have difficulty understanding task elements^46^, the observed brain changes may help them orient their attention to task features in the absence of therapist feedback.

Children with DCD demonstrated transfer of motor learning to other motor tasks after CO-OP^13^, which may be a result of orienting their attention to salient cues and guiding their behavior using their acquired self-regulation skills. This process demands greater attentional resources, which may be supported by increased within-network functional connectivity in the DAN. On the other hand, the left precentral gyrus is associated with action, perception, and cognition^79^, and in particular, with sustained attention for children with DCD^48^. It is also part of the primary motor cortex specifically associated with task execution^80^. Therefore, its improved functional connectivity with the DAN may regulate attentional demands and, then, facilitate motor execution, explaining the transfer of learning to other motor tasks in the follow-up analysis.

The DAN couples with the DMN during goal-directed learning, in order to provide both goal-directed cognition and internally-directed attention required for self-regulation^81,82^; thus, maintained strengthened connectivity of DMN and increased within-network functional connectivity of the DAN three months after the CO-OP intervention enable children with DCD to continue using their acquired skills and transfer their motor learning to other tasks.

### Movement quality predicts functional connectivity

Increased functional connectivity between the DMN and the right cerebellar lobules I–IV is associated with higher movement quality in children with DCD after intervention. Lobules I–IV of the cerebellum is part of the sensorimotor network^83–85^. Children with DCD have impaired functional connectivity between these two networks, leading to difficulty in allocating appropriate attentional allocation to sensorimotor tasks^53^. Since improved motor function after CO-OP seems to be related to improved self-regulation and strengthened functional connectivity of associated brain regions, the intervention may indirectly affect the functional connectivity between the DMN and the sensorimotor network to direct attention to salient features of the motor task. Increased functional connectivity of the DMN with task-related brain regions (in this case sensorimotor network) can also indicate automatization of learning^65^.

In contrast, for children with a dual diagnosis of DCD and ADHD, motor quality was predicted by strengthened functional connectivity of the frontoparietal network and seven regions of the cerebellum (right dentate, right and left lobule VI, right lobule VIIIa and VIIIb, right crus II, and left interpose). All of these regions are known to be part of the sensorimotor network, as well as various cognitive, frontoparietal, ventral attention, and salience networks^83,86^. Consistent with our findings, motor learning modulates functional connectivity of frontoparietal and cerebellar resting networks^87^. These findings suggest that, despite improved motor performance after CO-OP intervention, children with DCD and ADHD rely on functional networks that have been previously shown to be impaired^50^ to execute their motor tasks. Moreover, automatization, which is accompanied by decreased functional connectivity of the frontoparietal network^82^, did not occur in children with DCD + ADHD, and they continued to rely on attentional resources to perform motor tasks. Taken together, we infer that CO-OP has not affected the underlying cognitive determinants of motor learning, such as self-regulation and attention-regulation, in children with dual diagnoses of DCD and ADHD.

## Limitations

In this study, all children were from a similar geographic area which could reduce the generalizability of our results to a broader population of children with DCD ± ADHD; however, we believe they are representative of the clinical profile of children with DCD ± ADHD. Another limitation is that the follow-up analyses included a small sample of participants, reducing the analysis power; however, this is still the largest cohort to report longitudinal data following intervention in children with DCD ± ADHD. As we were not able to control for participants’ visual stimulus and whether the children’s eyes were open or closed during scans, we necessarily had to exclude visual networks from the analysis. Future studies should aim to address this limitation and perform further analysis using network analysis or graph theory to build upon our study results.

## Conclusion

Children with DCD showed increased functional connectivity of DMN with the pACC and improved motor skills after CO-OP intervention. This network is associated with self-, emotion-, and attention-regulation, which supports the hypothesis that self-regulation mediates motor learning^58^. Further, these brain changes were maintained three months after the completion of the intervention. Over the three months after the intervention, children with DCD also developed greater functional connectivity between the DAN and the precentral gyrus, which may be due to increased attentional demands during task execution in the absence of the therapist. These results provide the first line of neuroscientific evidence to show that CO-OP induces behavioral and neural changes in children with DCD that are maintained for at least three months. Although children with a dual diagnosis of DCD and ADHD did not show brain changes or transfer of motor learning to other tasks, these children showed improved motor skill acquisition after CO-OP intervention. As such, we recommend that pediatricians consider referring children with DCD (with and without ADHD) for CO-OP intervention to improve their motor skills.
